# A red yeast rice-olive extract supplement reduces biomarkers of oxidative stress, OxLDL and Lp-PLA_2_, in subjects with metabolic syndrome: a randomised, double-blind, placebo-controlled trial

**DOI:** 10.1186/s13063-017-2058-5

**Published:** 2017-07-03

**Authors:** Nina Hermans, Anastasia Van der Auwera, Annelies Breynaert, Annelies Verlaet, Tess De Bruyne, Luc Van Gaal, Luc Pieters, Veronique Verhoeven

**Affiliations:** 1 Natural Products and Food - Research & Analysis (NatuRA), Department of Pharmaceutical Sciences, University of Antwerp, Universiteitsplein 1, Antwerp, Belgium; 2 Department of Endocrinology, Diabetology and Metabolism, Antwerp University Hospital, Wilrijkstraat 10, 2650 Edegem, Belgium; 3 The academic centre for primary and interdisciplinary care, Faculty of Medicine and Health Sciences, University of Antwerp, Universiteitsplein 1, Antwerp, Belgium

**Keywords:** Metabolic syndrome, Red yeast rice, Olive, Oxidative stress

## Abstract

**Background:**

*Metabolic syndrome* (MetS) refers to clustered cardiovascular risk factors (abdominal obesity, pre-diabetes, high blood pressure, dyslipidaemia). Therapies targeting oxidative stress may delay progression to atherosclerosis and diabetes. We investigated the anti-oxidative effect of a supplement combining red yeast rice and olive extract in patients with MetS.

**Methods:**

A double-blind, placebo-controlled, randomised trial was conducted with 50 patients with MetS as defined by National Cholesterol Education Program Adult Treatment Panel III criteria. Forty-nine subjects randomly assigned to red yeast rice-olive extract (RYR-olive extract; 10.82 mg of monacolins and 9.32 mg of hydroxytyrosol per Cholesfytolplus capsule) or placebo completed the 8-week trial. Whereas effects on cardiovascular risk parameters of MetS have been reported recently, the observed significant 20% increase in oxidised low-density lipoprotein (OxLDL) prompted us to investigate other oxidative stress-related parameters: malondialdehyde (MDA), lipoprotein-associated phospholipase A_2_ (Lp-PLA_2_) and 8-hydroxy-2′-deoxyguanosine (8-OHdG). Statistical calculations included univariate quantitative analysis, multivariate linear regression and correlation analysis.

**Results:**

The updated results indicate that an RYR-olive extract supplement significantly reduced Lp-PLA_2_ by 7% (*p* < 0.001), but it failed to show a significant decrease in plasma MDA and 8-OHdG (*p* > 0.05). Reductions in OxLDL (20%) and Lp-PLA_2_ (7%) were associated with each other (*r* = 0.740, *p* < 0.001).

**Conclusions:**

RYR-olive extract significantly reduced Lp-PLA_2_ in correlation with the marked reduction in plasma OxLDL, which may lead to a reduced risk for cardiovascular disease in patients with MetS.

**Trial registration:**

ClinicalTrials.gov identifier: NCT02065180. Registered on 13 February 2014.

**Electronic supplementary material:**

The online version of this article (doi:10.1186/s13063-017-2058-5) contains supplementary material, which is available to authorized users.

## Background

‘Syndrome X’, now called the *metabolic syndrome’* (MetS), refers to the clustering of cardiovascular risk factors, including abdominal obesity, pre-diabetes, high blood pressure and dyslipidaemia. A combination of several of these conditions increases the risk for the development of chronic diseases such as type 2 diabetes mellitus and cardiovascular diseases. To date, the prevalence of MetS is increasing at a disturbing rate, and within the context of its proven association with cardiovascular disease, the leading cause of mortality in the modern world, more research is needed to unravel new therapeutic approaches [1].

Oxidative stress is involved in clinical manifestations related to MetS [2–4]. Higher plasma concentrations of malondialdehyde (MDA) [5, 6] and 8-iso-prostaglandin F2α [7, 8] were associated with MetS. Also, increased oxidised low-density lipoprotein (OxLDL) levels—OxLDL is a very important biomarker of oxidative lipid damage [9], given its pro-inflammatory and pro-atherogenic activity [10]—are observed in MetS. [11, 12]. In recent years, there has also been growing interest in lipoprotein-associated phospholipase A_2_ (Lp-PLA_2_), a key enzyme that catalyses OxLDL hydrolysis, thus producing pro-inflammatory mediators such as lysophosphatidylcholine. Increased plasma Lp-PLA_2_ activities have been reported in patients with MetS [9, 10], and Lp-PLA_2_ is now identified as a good determinant of MetS [13]. Moreover, the association of this enzyme with coronary heart disease risk has been shown in different studies [14, 15]. Therefore, assessment of this biomarker is important in risk assessment of MetS as well as in the evaluation of a potential treatment. In early stages of type 2 diabetes mellitus, a positive correlation is observed between OxLDL and Lp-PLA_2_ [16].

In cases of failure of lifestyle optimisation, treatment of MetS comprises a combination of drugs against particular aspects of MetS, including anti-hypertensive therapy and treatment of dyslipidaemia.

Novel health strategies are needed, however. In particular, anti-oxidant functional foods or food supplements may have beneficial effects on MetS. In this context, a double-blind, placebo-controlled, randomised trial of a commercially available food supplement combining red yeast rice (RYR) (*Oryza sativa* L. fermented by the yeast *Monascus purpureus* Went) and olive fruit (*Olea europaea* L.) extract was conducted in patients with MetS. The combination in this food supplement of red yeast rice and olive fruit extract (RYR-olive extract) was based on their health effects, which have been documented in the literature [17–25]. This has led to the approval of health claims by the European Food Safety Authority (EFSA) for both RYR and polyphenols in olive oil [20, 26]. Although the anti-oxidant effect of olive oil has been proven in many studies [17–21], which has led to the approval of the EFSA health claim, this claim has not been authorised yet for olive fruit extract.

Apart from the evaluation of the effect of this combination of olive fruit extract with RYR on clinical biomarkers of MetS, including waist circumference, lipid status and blood pressure, which have been published recently [27], this updated sub-analysis of the previous study was focused on its effect on biomarkers of oxidative stress. The effect on OxLDL levels has been reported previously [27] to correlate with the observed decreased LDL levels. Because MetS increases the risk of cardiovascular disease and a significant reduction of OxLDL has been observed, our extended study was focused on the assessment of specific biomarkers of oxidative damage to lipids and lipoproteins, including plasma MDA and Lp-PLA_2_. Results for OxLDL have been linked to Lp-PLA_2_ levels. In addition, 8-hydroxy-2′-deoxyguanosine (8-OHdG), a biomarker of oxidative damage to DNA, was assessed in urine. To our knowledge, this is the first study including an assessment of the effect of RYR and olive extract on oxidative stress in patients with MetS.

## Methods

### Study protocol and patient population

The study protocol and patient population description were published recently [27]. Briefly, a double-blind, placebo-controlled, randomised trial was conducted with 50 patients enrolled at the University of Antwerp. The sample size calculation was based on SD values in previous studies, and we determined that a minimum of 40 patients would be needed to establish an LDL cholesterol reduction of 15% between the intervention and control groups (power 0.80, significance level 0.05). The LDL difference was chosen because it is the primary target of statins. Participants had MetS according to National Cholesterol Education Program Adult Treatment Panel III criteria [28], which define MetS as a constellation of metabolic abnormalities requiring at least three of the following: increased waist circumference (>120 cm for men, >88 cm for women), elevated serum triacylglycerol level (≥150 mg/dl), reduced high-density lipoprotein cholesterol (<40 mg/dl for men, <50 mg/dl for women), increased blood pressure (≥130/85 mmHg or drug treatment for hypertension) and increased fasting blood glucose level (≥100 mg/dl).

Participants were excluded from the trial if they were under 18 years of age, treated with cholesterol-lowering drugs, had a pre-existing condition of chronic inflammatory diseases, had a triacylglycerol level >400 mg/dl or had a desire for pregnancy during the study. Factors possibly affecting oxidative stress status were registered by means of a questionnaire which included a detailed registration of dietary habits, physical exercise, smoking, menopausal status and perceived level of stress in daily life. Potential recruits taking cholesterol-lowering food products needed to stop these before the start of the study (wash-out period of 2 weeks).

We used a commercially available food supplement of red yeast rice and olive fruit extract (RYR-olive extract; Tilman, Baillonville, Belgium) and a placebo which looked exactly the same as the original product. Participants were instructed to take one capsule every evening for 8 weeks. No dietary measures were imposed.

Participants were randomised to the control (*n* = 24) or intervention (*n* = 26) group by random generation of even (intervention) and uneven (control) numbers by means of a computer program (www.random.org). No stratification for age, sex or cholesterol level was performed. Study groups were blinded to participants and researchers. A written informed consent form was obtained from all potential recruits at the time of inclusion.

Fasting blood and urine samples were drawn at the beginning and at the end of the study. For analyses of biomarkers of oxidative stress, blood samples were collected in ethylenediaminetetraacetic acid-coated tubes (BD Benelux, Erembodegem, Belgium) at the beginning and at the end of the study, and plasma was separated by centrifugation (2000 × *g* for 10 minutes at 4 °C) and stored at −80 °C. Anti-oxidant outcome measures, MDA, OxLDL, Lp-PLA2 and 8-OHdG levels were determined. The study was approved by the ethical review board of the Antwerp University Hospital, and it is registered with ClinicalTrials.gov (NCT02065180).

### RYR-olive extract supplement

A commercially available food supplement (Cholesfytolplus; Tilman) has been used in comparison with a placebo that looked similar to the original product. Each capsule of the RYR-olive extract supplement consisted of RYR containing 10.82 ± 0.84 mg of monacolin K (of which 5.88 ± 0.46 mg was lovastatin), olive fruit extract containing 9.32 ± 0.54 mg of hydroxytyrosol, as determined by high-pressure liquid chromatography (HPLC) [27], and the same excipient mixture as in the placebo capsule. The placebo consisted of an excipient mixture of talcum, magnesium stearate, microcrystalline cellulose, colloidal anhydrous silica and tricalcium phosphate.

### Analysis of biomarkers of oxidative stress

#### MDA

Oxidative damage caused by lipid peroxidation was determined in plasma using a previously optimised and validated HPLC fluorescence detection method [29]. Briefly, 50 μl of sample was mixed with 25 μl of 1% butylated hydroxytoluene, 250 μl of 1.22 M phosphoric acid, 425 μl of HPLC-grade water and 250 μl of 0.67% thiobarbituric acid. After incubation at 95 °C for 40 minutes and protein precipitation, samples were analysed on an Agilent 1260 HPLC system (Agilent Technologies, Diegem, Belgium) with a Jasco FP-1520 fluorescence detector (Jasco Benelux, Utrecht, The Netherlands) at 532 nm.

#### OxLDL

Plasma OxLDL was analysed using an enzyme immunoassay [27].

#### Lp-PLA_2_

Plasma Lp-PLA_2_ activity was measured at baseline for all patients, and a subgroup of 26 patients with moderate to high Lp-PLA_2_ levels (>151 nmol/ml/minute) was selected for follow-up measurements at 8 weeks of treatment. Lp-PLA_2_ activity was determined at RCI Laboratories (Ghent, Belgium) by performing an automated colorimetric activity test (diaDexus PLAC activity test; Diazyme Laboratories, San Diego, CA, USA) on a clinical chemistry analyser using a colorimetric platelet-activating factor analogue substrate (with a nitrophenol label) that is converted upon hydrolysis by the Lp-PLA_2_ enzyme. Hydrolysis of the colorimetric substrate was monitored by observing changes in visible absorbance over time (nmol/minute ml) by using a standard curve for nitrophenol absorbance.

#### 8-OHdG

Urinary 8-OHdG, a biomarker of oxidative DNA damage, was analysed by using an enzyme-linked immunosorbent assay (ELISA) kit (NWLSS^TM^ Urinary 8OHdG ELISA Northwest Life Science Specialties, Vancouver, WA, USA). This assay uses a competitive ELISA wherein a murine monoclonal antibody to 8-OHdG and urine sample are added to a microtiter plate which has been pre-coated with 8-OHdG. Sample 8-OHdG competes with plate-bound 8-OHdG for binding with the antibody. Practically, 50 μl of urine (or standard or control) was added to the 8-OHdG-coated well. A quantity of 50 μl of murine anti-8-OHdG monoclonal antibody was added to the wells, except for blank wells. After incubation (1 h, 37 °C), the plate was emptied and washed three times with 250 μl of PBS. Next, 100 μl of anti-murine antibodies conjugated with horseradish peroxidase was added, incubated (1 h, 37 °C), emptied and washed with PBS. After that, 100 μl of 3,3′,5,5′-tetramethylbenzidine was added, followed by incubation for 15 minutes at room temperature in the dark. Before measuring the absorbance at 450 nm using a microplate reader (Synergy Mx; BioTek, Winooski, VT, USA), 100 μl of phosphoric acid (1 M) was added. The urinary concentrations of 8-OHdG were expressed as nanograms per milligram of creatinine. Urinary creatinine levels were analysed by Creatinine Microplate Assay CR01 (Oxford Biomedical Research, Rochester Hills, MI, USA).

### Statistical analyses

All data are presented as mean ± SEM. Statistical calculations were performed using SPSS software (SPSS Inc., Chicago, IL, USA) and included univariate quantitative analysis (independent samples *t* test, chi–square test, Fisher’s exact test), multivariate linear regression, and correlation analysis (Pearson’s correlation coefficient).

## Results

**Fig. 1:**
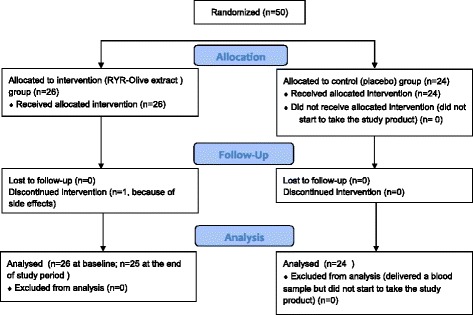
Consolidated Standards of Reporting Trials (CONSORT) flow diagram. *RYR* Red yeast rice-olive extract [27]

Fifty participants complying with the inclusion criteria entered the study and were randomly allocated to the RYR-olive extract (*n* = 26) or placebo (*n* = 24) group [27]. The flow of patients through the study is shown in Fig. 1.

Baseline characteristics of the study population are reported elsewhere [27], and there were no differences in clinical parameters or in dietary and physical habits between the RYR-olive extract intervention and placebo groups. Daily consumption of this RYR-olive extract, containing 10.82 ± 0.84 mg of monacolin K and 9.32 ± 0.54 mg of hydroxytyrosol, resulted in a 24% decrease of LDL levels. Plasma OxLDL was significantly reduced in the RYR-olive extract-treated group (absolute difference −19.35 ± 4.43 U/L) compared with the placebo group (absolute difference 3.65 ± 2.35 U/L) (*p* < 0.001). A relative reduction of OxLDL of 20% was observed in patients receiving RYR-olive extract (*p* < 0.001) [27]. In the present study, the effects on biomarkers of oxidative stress were assessed. Baseline oxidative stress levels are depicted in Table 1.

**Table 1:** Baseline oxidative stress levels of the study population

Characteristic	RYR-olive extract (*n* = 26)	Placebo (*n* = 24)	*p* Value^a^
MDA, μM	0.405 (0.097)	0.395 (0.077)	0.69
OxLDL, U/L	80.75 (34.54)	69.10 (17.67)	0.16
Lp-PLA_2_, nmol/ml/minute	191.78 (21.80)	192.89 (28.09)	0.91
8-OHdG, ng/mg creatinine	10.85 (4.33)	14.15 (5.10)	0.03

*Abbreviations: Lp-PLA*_*2*_ Lipoprotein-associated phospholipase A_2_, *MDA* Malondialdehyde, *8-OHdG* 8-Hydroxy-2′-deoxyguanosine, *OxLDL* Oxidised low-density lipoprotein, *RYR* Red yeast rice

Values are expressed as mean ± SD

^a^Independent samples *t* test, chi-square test or Fisher’s exact test

**Fig. 2:**
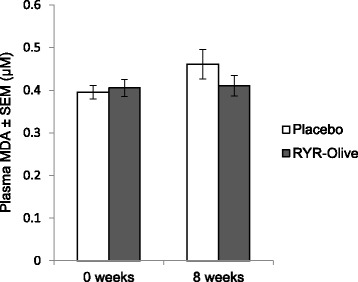
Plasma malondialdehyde (MDA) at baseline and 8 weeks in *red* yeast rice (RYR)-olive and placebo groups (*n* = 49)

No significant difference was found in plasma MDA levels between the RYR-olive extract and placebo groups. Absolute and relative mean differences in the RYR-olive extract intervention group were 0.01 ± 0.02 μM and 2.35 ± 3.69%, respectively, and in the placebo group, the corresponding values were 0.07 ± 0.03 μM and 17.10 ± 6.81%, respectively (after 8 weeks). Figure 2 depicts mean MDA concentrations in both groups at baseline and after 8 weeks.

**Fig. 3:**
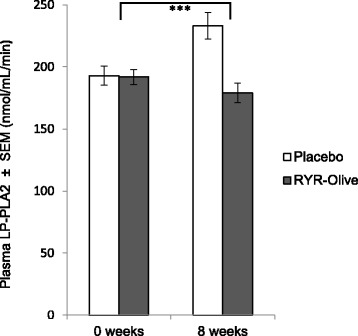
Plasma lipoprotein-associated phospholipase A_2_ (Lp-PLA_2_) at baseline and 8 weeks in red yeast rice (RYR)-olive and placebo groups. ***p < 0.001 mean difference between placebo and intervention groups (*n* = 26)

In 26 patients, baseline measurements of plasma Lp-PLA_2_ were moderate to high (>151 nmol/ml/minute). After 8 weeks of the intervention, Lp-PLA_2_ was specifically screened in this subgroup. Patients in the RYR-olive extract-treated group (*n* = 13) had significantly reduced Lp-PLA_2_ levels after 8 weeks (−12.72 ± 5.44 nmol/ml/minute) of supplementation compared with those in the placebo group (*n* = 13; 40.16 ± 6.12 nmol/ml/minute) (*p* < 0.001). The mean reduction of Lp-PLA_2_ in the intervention group was 6.69%. In the placebo group, no reduction of Lp-PLA_2_ could be observed in any patient. The results of Lp-PLA2 screening are depicted in Fig. 3.

**Fig. 4:**
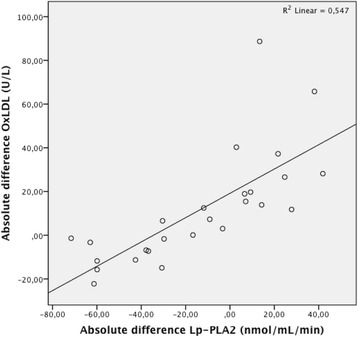
Correlation between oxidised low-density lipoprotein (OxLDL) and lipoprotein-associated phospholipase A_2_ (Lp-PLA_2_) plasma levels

In the subgroup of patients with high Lp-PLA_2_ (*n* = 26), a Pearson correlation test was conducted to investigate a possible correlation between before-and-after differences of OxLDL [27] and before-and-after differences in Lp-PLA_2_. A positive correlation was observed between these two parameters (*r* = 0.740, *n* = 26, *p* < 0.001) (Fig. 4).

**Fig. 5:**
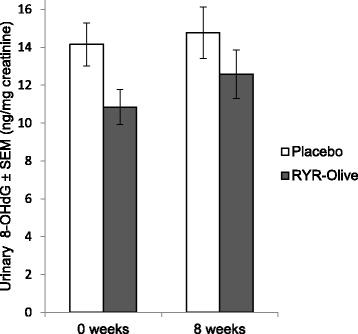
Urinary 8-hydroxy-2′-deoxyguanosine (8-OHdG) at baseline and 8 weeks in *red* yeast rice (RYR)-olive and placebo groups (*n* = 49)

With regard to urinary 8-OHdG, a biomarker of oxidative DNA damage, no significant difference was observed. Figure 5 shows mean urinary 8-OHdG levels in both groups at baseline and after 8 weeks.

## Discussion

Current evidence suggests that oxidative stress may be an early manifestation which plays a central role in the development of MetS. Thus, therapeutic approaches which target oxidative stress may delay disease progression [4, 30–32].

To our knowledge, this is the first study involving an investigation of the effect of a preparation of RYR and olive fruit extract on biomarkers of oxidative stress. Apart from a significant reduction of total cholesterol, LDL, triacylglycerol and OxLDL levels, which have been reported previously [27], the 2-month administration of this RYR-olive fruit extract supplement resulted in a significant decrease in plasma Lp-PLA_2_, a biomarker of oxidative damage to lipoproteins. After 8 weeks of treatment, OxLDL was reduced by 20% and Lp-PLA_2_ by 7% in the RYR-olive extract-treated group. In recent years, many studies have shown the contribution of these biomarkers to the inflammation process and the risk of coronary disease. OxLDL has been shown to be an important factor in atherosclerosis initiation and development [33, 34]. Other recent studies have shown that circulating Lp-PLA_2_ activity is associated with risk of coronary disease and vascular mortality [14, 15, 35]. Recently, the association of OxLDL and Lp-PLA_2_ has been proven both ex vivo in human atherosclerotic lesions and in vivo in patients with MetS [9, 33].

In this study, the reduction of Lp-PLA_2_ was positively correlated with that of OxLDL [27] after 8-week treatment with the RYR-olive fruit extract in the subgroup of patients with high Lp-PLA_2_ activity (>151 nmol/ml/minute). This is an interesting result which needs to be studied further in long-term follow-up trials. Given the recent associations of high Lp-PLA_2_ activity in plasma and cardiovascular risk, supplementation of this anti-oxidant supplement in patients with MetS, specifically those with increased Lp-PLA_2_ activity, might reduce the manifestation of severe cardiovascular events.

The protective effects of olive oil polyphenols against oxidative damage to lipids, particularly oxidation of LDL, have been shown in different recent studies, and dose-effect relationships have been established [17–19, 21]. Because OxLDL plasma levels and Lp-PLA_2_ plasma activity in the food supplement-treated group decreased, the anti-oxidant effects of this supplement are confirmed in the present study. Although the observed anti-oxidant effects were at least in part due to the hydroxytyrosol-rich olive fruit extract, it was not possible with the present study design to unambiguously assign the anti-oxidant activity to the olive fruit extract. The product also contained RYR delivering 10.84 ± 0.84 mg of monacolin K (of which 5.88 ± 0.46 mg was in lovastatin lactone form). This is an important aspect because researchers in recent studies also reported plasma OxLDL level- and Lp-PLA2 activity-reducing effects of certain statins, such as simvastatin and pravastatin [14, 34]. Although those trials were longer and the doses of the synthetic statins administered were higher than the monacolin K delivered by the RYR in the present study, it is possible that the monacolin K contributed to the anti-oxidant effects observed here. To dedicate the biologic effects to the specific components in the food supplement, and to investigate possible synergistic effects of combining both, a trial should be conducted to compare the effect of an RYR extract on one hand and an olive fruit extract (both with comparable composition to the extracts tested in this trial) on the other, on these biomarkers of oxidative stress.

The short follow-up period of 8 weeks is also a limitation of the study. A follow-up study over a longer period should be conducted to draw definite conclusions on the effect of the reduction of these biomarkers of oxidative stress on the reduction of cardiovascular disease occurrence.

MDA, another biomarker of oxidative damage to lipids, did not change significantly, which has been observed regularly in early oxidative stress-related disease stages. In general, MDA values obtained tend to differ in function of the experimental set-up in animal studies, as well as in the severity of the observed pathology and analytical technique used for MDA quantification. This is apparent both in observational studies comparing disease and control groups and in interventional trials investigating a potential therapy. Armutcu and co-workers, using a spectrophotometric method, did observe significantly higher MDA values in subjects with MetS than in a healthy control group [5]. Our previous research, however, demonstrated that MDA quantification by HPLC is preferable, thus avoiding possible interfering factors [29].

In animal models, a clearly developed pathology was found to be important to observing significant differences in plasma MDA because a diabetic rat model and an atherosclerotic rabbit model did show increased plasma MDA values (indicating increased systemic oxidative lipid damage), whereas this was not seen in vitamin E-deficient rats [36].

Other biomarkers of oxidative lipid damage have been reported to differ in patients with MetS. Thiobarbituric acid reactive substances (TBARS) have been found to be increased in obese patients with MetS (mean BMI 33 kg/m^2^) [6]. In that previous study, BMI (33 kg/m^2^) was more elevated than in our population (BMI 27.5–27.8 kg/m^2^), and TBARS levels were determined spectrophotometrically. Plasma F2-isoprostanes were positively associated with most components of MetS in a recent report by Black et al. [37]. In that same report, however, 8-OHdG, a biomarker of oxidative damage to DNA, did not show significant changes. In our study, a significant change in 8-OHdG was also not observed. Parameters of oxidative lipid damage are more likely to be affected in MetS, involving altered lipid metabolism, than biomarkers of DNA damage. This reflects two distinct pathways of oxidative damage that are not necessarily closely inter-related and confirms the importance of measuring multiple markers of oxidative stress [37].

Assessment of both biomarkers MDA and 8-OHdG after supplementation over a longer period merits attention to draw definite conclusions regarding these parameters. Given the increased risk of major cardiovascular events among patients with MetS, the present data regarding the anti-oxidant effects of an RYR-olive fruit extract, as well as its effects on blood lipid parameters and blood pressure as reported in our recent publication [27], clearly demonstrate the value of this food supplement in preventing deterioration of metabolic abnormalities.

## Conclusions

After 8 weeks of treatment of subjects with MetS in a double-blind, placebo-controlled, randomised trial, daily intake of a RYR-olive fruit extract supplement containing 10.82 ± 0.84 mg of monacolin K and 9.32 ± 0.54 mg of hydroxytyrosol as bioactive compounds resulted in a 7% decrease of Lp-PLA_2_ and correlated with a 20% decrease of plasma OxLDL. The reduction in these oxidative stress biomarkers may lead to a reduced risk of cardiovascular disease in patients with MetS in the longer term. Future investigations should be focused on the long-term anti-oxidant effects of this supplement and the prevention of cardiovascular events.

### CONSORT statement

This paper adheres to the Consolidated Standards of Reporting Trials (CONSORT) guidelines. Compliance with these guidelines is demonstrated in Additional file 1.

## Additional file

Additional file 1: Consolidated Standards of Reporting Trials (CONSORT) checklist. (DOC 219 kb)

## Abbreviations

CONSORT: Consolidated Standards of Reporting Trials
EFSA: European Food Safety Authority
ELISA: Enzyme-linked immunosorbent assay
HPLC: High-pressure liquid chromatography
LDL: Low-density lipoprotein
Lp-PLA_2_: Lipoprotein-associated phospholipase A_2_
MDA: Malondialdehyde
MetS: Metabolic syndrome
8-OHdG: 8-Hydroxy-2′-deoxyguanosine
OxLDL: Oxidised low-density lipoprotein
RYR: Red yeast rice
TBARS: Thiobarbituric acid reactive substances
